# Draft genomes of halophilic Archaea strains isolated from brines of the Carpathian Foreland, Poland

**DOI:** 10.7150/jgen.82493

**Published:** 2023-04-10

**Authors:** Jakub Lach, Dominik Strapagiel, Agnieszka Matera-Witkiewicz, Paweł Stączek

**Affiliations:** 1 Department of Molecular Microbiology, Faculty of Biology and Environmental Protection, University of Lodz, Lodz, Poland; 2 Biobank Lab, Department of Oncobiology and Epigenetics, Faculty of Biology and Environmental Protection, University of Lodz. Lodz, Poland; 3 Screening of Biological Activity Assays and Collection of Biological Material Laboratory, Wroclaw Medical University Biobank, Faculty of Pharmacy, Wroclaw Medical University, Wroclaw, Poland

## Abstract

Halophilic Archaea are a unique group of microorganisms living in saline environments. They constitute a complex group whose biodiversity has not been thoroughly studied. Here, we report three draft genomes of halophilic Archaea isolated from brines, representing the genera of *Halorubrum*, *Halopenitus,* and *Haloarcula*. Two of these strains, Boch-26 and POP-27, were identified as members of the genera *Halorubrum* and *Halopenitus*, respectively. However, they could not be assigned to any known species because of the excessive difference in genome sequences between these strains and any other described genomes. In contrast, the third strain, Boch-26, was identified as *Haloarcula hispanica*. Genome lengths of these isolates ranged from 2.7 Mbp to 3.0 Mbp, and GC content was in the 63.77%-68.77% range. Moreover, functional analysis revealed biosynthetic gene clusters (BGCs) related to terpenes production in all analysed genomes and one BGC for RRE (RiPP recognition element)-dependent RiPP (post-translationally modified peptides) biosynthesis. Moreover, the obtained results enhanced the knowledge about the salt mines microbiota biodiversity as a poorly explored environment so far.

## Introduction

Archaea as representatives of an extremely diverse group of organisms are present in almost all types of environments worldwide. They can be found in the guts of humans and animals, in harsh habitats such as salt mines or hot springs and in food products 1-3. Over the years, research on Archaea has been very limited due to the limited availability of methods for studying these microorganisms. However, in the last decade, significant progress has been made in understanding the taxonomic and metabolic diversity of this group of organisms 4. It is associated with the development of research methods related to high-throughput DNA sequencing and metagenome analyses, which provided greater insight into the microbial “dark matter” 5. Metagenomic analysis of various environments has allowed the identification of new taxonomic groups among Archaea, such as Asgard archaea or DPANN 6,7. Research is currently underway to develop Archaea cultivation methods, which remains a significant challenge, and for many taxa, pure cultures have still not been isolated 8.

Archaea constitute a significant part of extremophilic microorganisms that are adapted to survive in conditions inaccessible to the most known microbes 4. This group also includes halophilic microorganisms living in high-salinity environments. Most of them can be found in the classes *Halobacteria* and *Methanomicrobia* belonging to the phylum *Euryarcheota* 9. Due to their ability to live in high salinity conditions, these microorganisms can be used for industrial purposes in bioprocesses 10,11. Bioproducts such as ectoine, polyhydroxyalkanoate (PHA) or extremozymes produced by halophiles found applications in many areas of biotechnology including the production of polyunsaturated fatty acids, biopolymers and osmoprotectants 11,12. Research was also conducted to identify and characterize compounds that may find application in medicine, as was the case with Actinomycin C2, Streptomonomicin or halocins 13.

The microbiota of salt mines and brines is still not very well explored. Only a few strains of microorganisms have been isolated from this kind of habitat, and only a few metagenomes have been sequenced. Such species, like *Halorhabdus rudnickae*, *Halorubrum trueperi* and *Halorubrum amylolyticum,* were identified for the first time in samples collected from such environments 14-16. Metagenomic analysis for salt mines has been performed so far for the Karak Salt Mine, Pakistan 2. Despite the research carried out, there are still significant gaps in the knowledge about halophiles inhabiting salt mines and brines what determines the need for a thorough study of such environments for a better understanding their biodiversity and to discover possible applications of extremophiles.

The salt mine in Bochnia (southern Poland) is an example of an environment that, due to its uniqueness, can be a habitat for many previously unknown microbial strains and which has not yet been thoroughly investigated. It was established in the 13th century and was actively exploited until 1990. It means that it was one of the oldest and longest-exploited salt mines in Europe. Due to its long history and unique character, it was placed on the UNESCO World Heritage List 17. It was first established in a fragment of the marine sediments of the Miocene salt-bearing formation. Currently, the historic mine consists of nine post-mining galleries reaching 350 m below the surface, and a significant part of the excavations is open to the public 18. Since the end of salt mining, the activity of the Bochnia salt mine has changed its character and focused on tourism, recreation, and health protection.

In this paper, we report three genomes of Archaea isolated from brines collected from the Bochnia Salt Mine, located in southern Poland, near the city of Kraków. The analysed strains were characterized in terms of taxonomy and functionality, thus enriching the knowledge of the microbiota of the Bochnia Salt Mine.

## Materials and Methods

Three strains of halophilic Archaea were isolated from brines collected in the Bochnia Salt Mine (49°58′09″N 20°25′03″E). Isolation of strains was carried out in accordance with the methodology used in earlier studies by Albuquerque et al. 15. All strains were cultured on plates containing halobacteria medium (DSMZ 372) with 25% (w/v) NaCl concentration. The culture temperature was 37 °C for the strains *Halorubrum* Boch-26 and *Halopenitus* POP-27, and 28°C for the strain *Haloarcula hispanica* Boch-4. Genomic DNAs were extracted using the QIAamp DNA Mini Kit (Qiagen, Hilden, Germany).

For each strain, 1 ng of high-quality genomic DNA was used for DNA library preparation. Nextera XT DNA sample preparation kit (Illumina Inc., San Diego, USA) was used for the preparation of paired-ends libraries. The libraries were sequenced in a 2 × 150 bp configuration using a NextSeq 500 instrument (Illumina, San Diego, USA). The quality of reads was checked with FastQC 19. Adaptors and low-quality sequences were trimmed out from the reads with Trim galore! v. 0.6.4 set on default parameters 20. For *de novo* genomes assembly, SPAdes v3.15.0 was used 21. Contigs shorter than 500 bp or with sequencing depths lower than 2 were removed from the assemblies. For quality control of assemblies, CheckM 22 and Quast 23 were used. Genome annotation was performed using Prokka v.1.14.0 24 and the eggNOG-mapper website v.5.0.0 25. Identification of biosynthetic gene clusters (BGCs) was performed with antiSMASH v.6.0.1 26. Taxonomic annotation of genomes was performed with gtdb-tk version 1.5.1 27. Type (Strain) Genome Server (TYGS) was used for phylogenomic analysis 28, and the resulting whole-genome sequence-based phylogenetic trees were visualized by iTOL 29. The d4 formula from TYGS was used to calculate dDDH. This metric was calculated as a sum of all identities found in high-scoring segment pairs (HSPs) divided by overall HSP length. For TYGS analysis, genomes of 29 reference strains were used. They are available in NCBI database under accessions: FOPZ00000000, VCNM00000000, JANHDN000000000, AOLR00000000, FNBO00000000, AOLS00000000, VCNL00000000, LOAJ00000000, SDJP00000000, FXTD00000000, LKIR00000000, NHPJ00000000, FNPC00000000, BMON00000000, BMPD00000000, AOLY00000000, AOJG00000000, AOLW00000000, AOLX00000000, AOJH00000000, BBJP00000000, AOJE00000000, RBWW00000000, CP002921-CP002923, AY596290-AY596298, CP001365-CP001367, HF582854, NSKC00000000, FNWU00000000. A phylogenetic tree for *gyrB* sequences was prepared in MEGA X software 30. The evolutionary distances between sequences were computed using the Kimura 2-parameter method.

All sequencing data are publicly available from the National Institutes of Health under WGS accessions JAQYWM000000000, JARANU000000000, JARANT000000000.

## Results and Discussion

The three draft genomes sequences of the strains analysed contained between 33 and 177 contigs. Their sizes ranged from 2.7-3.0 Mbps. Completeness of the genomes was assessed using CheckM and was between 95.97%-97.93%. Contamination of genomes has been estimated at a level of 0.0%-0.95%, and the GC content was in the 63.77%-68.77% range. General genome feature statistics have been summarized in Table 1.

**Figure 1:**
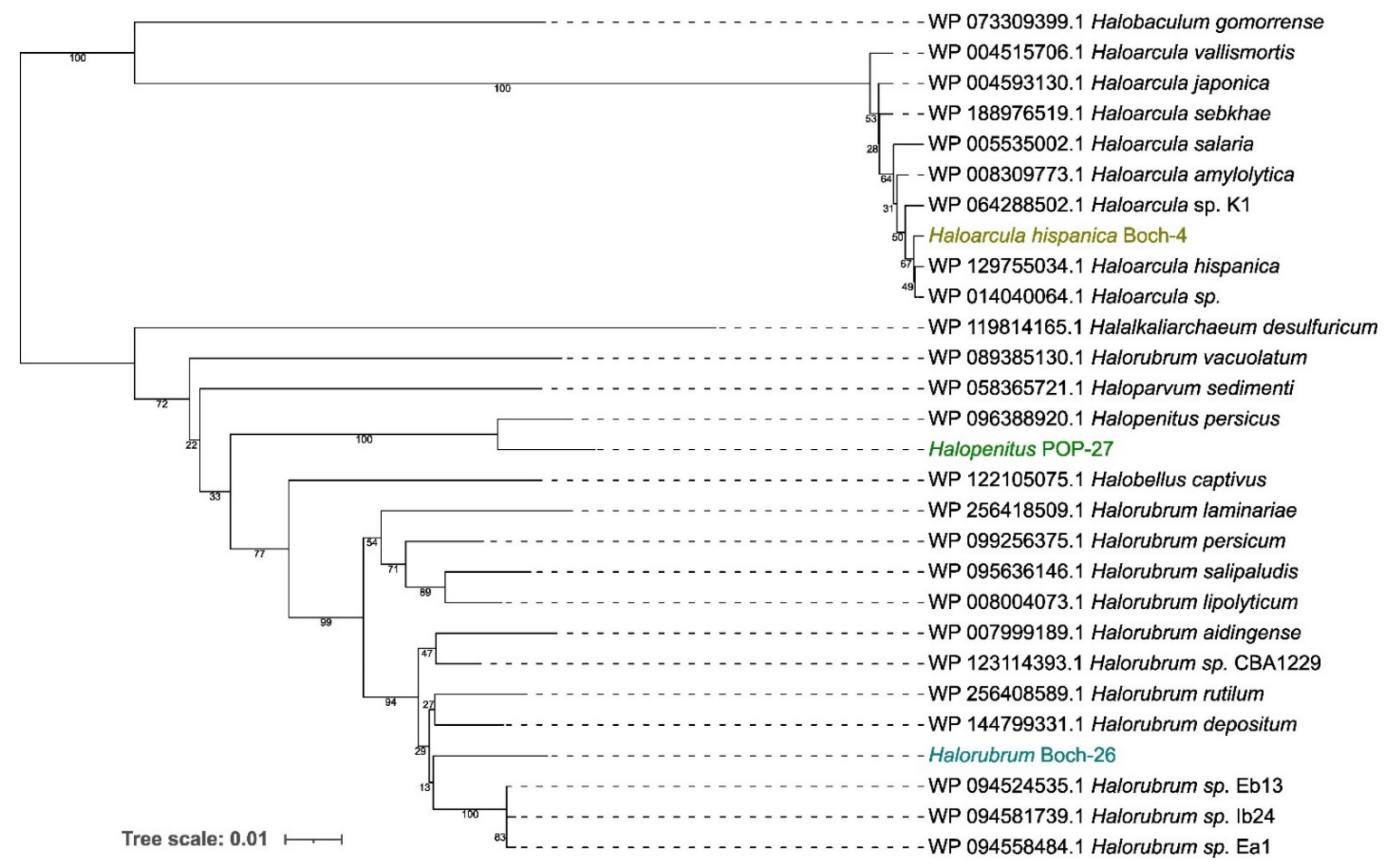
Phylogenetic tree based on the analysis of the *gyrB* sequences using the Neighbor-Joining method and showing the relationships between analysed strains and other *Chromohalobacter* and *Halomonas* strains.

**Figure 2:**
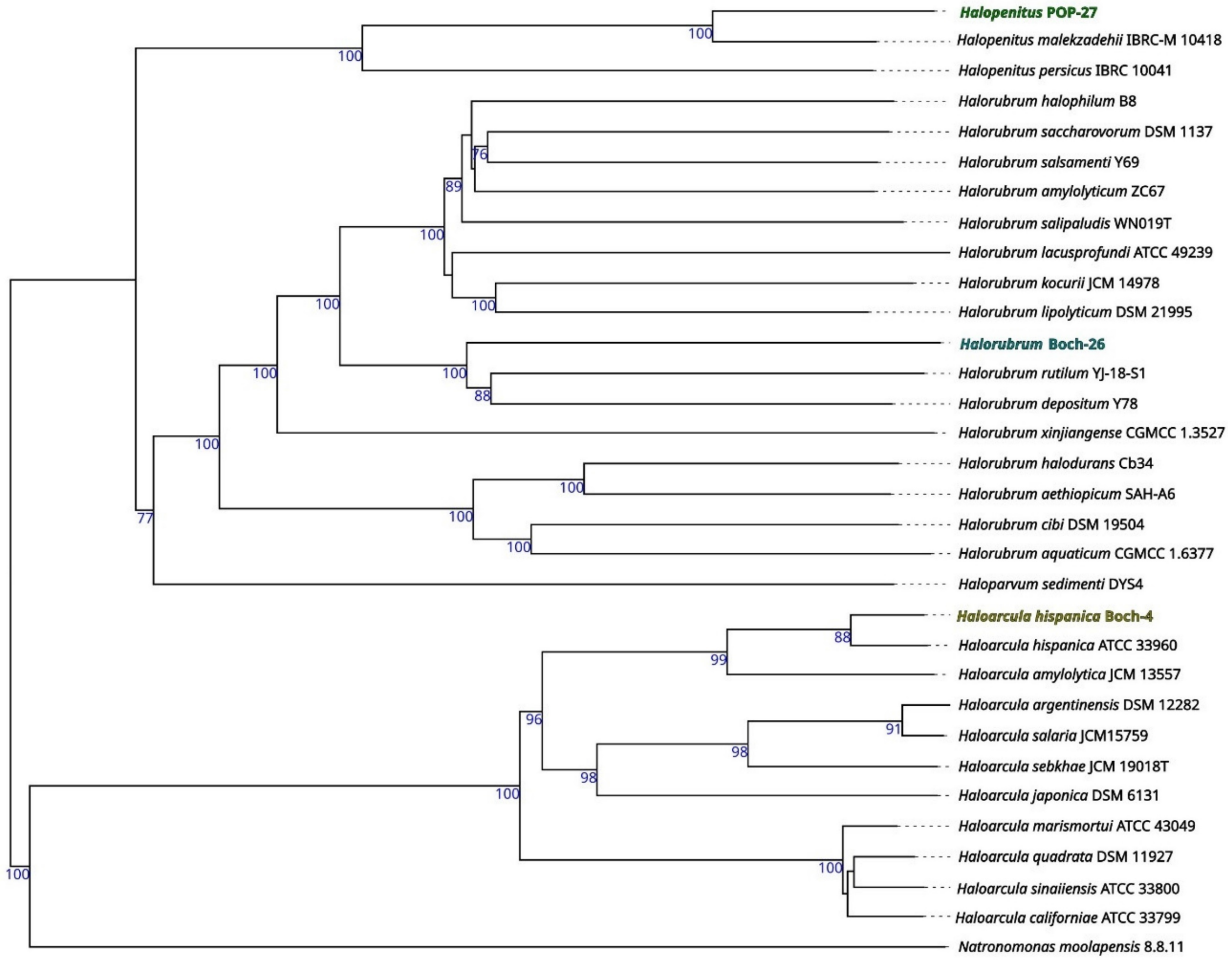
Whole-genome sequence-based phylogenetic tree build with Genome BLAST Distance Phylogeny approach (GBDP) by Type (Strain) Genome Server (TYGS).

Based on the genomic sequences, a preliminary taxonomic annotation was performed using the gtdb-tk tool. During the analysis, strains Boch-4 and POP-27 were assigned to *Haloarcula hispanica* and *Halopenitus malekzadehii,* respectively. For strain Boch-26, assignment to species was impossible due to the too high difference between the genome sequence of this strain and those of other known genomes collected in the GTDB (the Genome Taxonomy Database). In order to better describe the evolutionary relationships between the strains analysed and other closely related species, a phylogenetic tree based on sequences of the *gyrB* gene was prepared. The reconstructed phylogenetic tree partially confirmed the taxonomic classification performed with gtdb-tk (Figure 1). Strain Boch-26 was placed close to other *Halorubrum* species but did not form a single cluster with any of them. The same was true for strain Boch-4, which was placed in a single cluster with *Haloarcula hispanica.* In the case of strain POP-27, it was clustered together with *Halopenitus persicus,* but the sequence of the *gyrB* gene for *Halopenitus malekzadehii* was unavailable. To verify the results obtained from gtdb-tk and *gyrB* analysis, a phylogenetic tree based on the whole-genome sequences was prepared using the Genome BLAST Distance Phylogeny approach (Figure 2). The location of strains Boch-4 and Boch-26 on the reconstructed phylogenetic tree confirmed their taxonomic annotation. Strain Boch-4 clustered with *Haloarcula hispanica,* and the digital DNA-DNA hybridization (dDDH) value between these two genomes was 82.6%. Therefore, all analyses performed clearly indicated that strain Boch-4 belongs to the species* Haloarcula hispanica*. In the case of strain Boch-26, its taxonomic classification is also unequivocal. On a whole-genome sequence-based phylogenetic tree, this strain was placed among other *Halorubrum* species, but its distance from its other close relative, which was *Halorubrum depositum*, was too great to identify both strains as the same species. It was also confirmed by low dDDH between these two strains, which was 35.45%. The results for the POP-27 strain remain inconclusive. On the phylogenetic tree, this strain was located close to *Halopenitus malekzadehii* but dDDH between these two genomes was 59.2%, which proves that the genomes do not belong to the same species of microorganisms. The difference in the classification indications based on gtdb-tk and dDDH means that it is impossible to conclude unequivocally from the available data whether the POP-27 strain belongs to the species *Halopenitus malekzadehii*. Accordingly, the strain will be referred to as *Halopenitus* POP-27.

In order to characterise the functional profiles of the strains studied, a functional annotation of the genomes analysed was performed. The profiles obtained, based on eggNOG categories, are shown in Table 2. The *Halopenitus* strain POP-27 was characterised by a higher number of genes related to defence mechanisms, which amounted to 31 [1.27%] in this genome, while for the strains *Halorubrum* Boch-26 and *Haloarcula hispanica* Boch-4 these numbers were 15 [0.59%] and 27 [0.90%], respectively. The reverse was true for genes related to cell motility. In the *Halopenitus* strain POP-27, there was a significantly lower number of such genes (17 [0.70%]) than in the other two strains, *Halorubrum* Boch-26 (29 [1.15%]) and *Haloarcula hispanica* Boch-4 (41 [1.37%]). It is also significant that genes related to the biosynthesis of secondary metabolites were detected in all genomes. The secondary metabolites produced may be related to the adaptation of the studied strains to a harsh environment and may be of interest for industrial application. The content of these genes in particular strains was similar and amounted to 42 [1.66%], 37 [1.51%], and 43 [1.44%] for strains *Halorubrum* Boch-26, *Halopenitus* POP-27, and *Haloarcula hispanica* Boch-4, respectively. The results obtained were the basis for an attempt to identify the BGCs present in the analysed genomes. AntiSMASH was used to identify BGCs. Two BGCs associated with terpene production were identified in all three strains. These clusters were not identical between the strains however, each one was organised around genes coding phytoene synthase. It can therefore be assumed that these strains may produce carotenoids. Three BGCs were identified in the *Haloarcula hispanica* strain. As previously mentioned two of them were associated with the terpene production, and the last one with RRE (RiPP recognition element)-dependent RiPP (ribosomally synthesized and post-translationally modified peptides) biosynthesis. Both of BGC related to the terpene production were organised around genes coding phytoene synthase. This enzyme is usually involved in carotenoid production. The first BGC contained 23 genes, and antiSMASH identified two strains (*Haloarcula* sp. CBA1115 and *Haloarcula* sp. K1K1) containing BGCs in which 100% of the genes showed similarity to genes in the BGC analysed. The second BGC consisted of 20 genes, and BGCs in which 95% of the genes showed similarity to genes in the analysed BGC were found in strains such as *Haloarcula* sp. K1K1, *Haloarcula hispanica* ATCC33960, and *Haloarcula vallismortis* DSM3756. The third BGC associated with RiPP biosynthesis contained 19 genes, and antiSMASH did not identify any highly similar BGCs. The most similar BGC with 18% of similar genes was associated with lasso peptide production and was identified in *Halopiger xanaduensis* SH-6 plasmid. The BGC analysed contained genes related to the synthesis of B1 and B2 proteins involved in lasso peptides production. A gene encoding a kinase that plays an important role in lasso peptide synthesis of was also identified in this BGC.

Concluding, the draft genomes of three Archaea strains have been reported and characterised in this paper. These genomes represent a significant value due to the uniqueness and isolation of the environment from which they were extracted from other saline environments. Significant differences in genome sequences compared to other known halophiles provide a unique insight into the diversity of microorganisms that can inhabit salt mines. The analyses conducted indicate that two of the strains analysed, *Halorubrum* Boch-26 and *Halopenitus* POP-27, may belong to hitherto unknown species. However, confirmation of this observation requires further analyses in aspects related to the physiology and morphology of the isolates studied. The obtained results allow us to expand our knowledge about the biodiversity of the halophilic Archaea living in salt mine environments.

## Figures and Tables

**Table 1:** Genome features.

	Archaea		
	*Halorubrum* Boch-26	*Halopenitus* POP-27	*Haloarcula hispanica* Boch-4
Genome length (bp)	2 763 236	2 670 493	3 024 851
Number of contigs	177	33	34
Largest contig (bp)	66 542	332 978	285 480
GC content (%)	68.77	66.27	63.77
N50 (bp)	26 505	116 967	136 784
Number of CDSs	2 749	2 602	3 061
Number of rRNAs	1	1	1
Number of tRNAs	42	48	46
Number of repeat regions	0	0	0
Completeness (%)	95.97	97.84	97.93
Contamination (%)	0.95	0.38	0.0

**Table 2:** eggNOG categories of genes present in in the analysed genomes.

Class	Description	Archaea		
		*Halorubrum* Boch-26	*Halopenitus* POP-27	*Haloarcula hispanica* Boch-4
**Information storage and processing**				
J	Translation, ribosomal structure, and biogenesis	122 [4.82]	156 [6.38]	158 [5.29]
A	RNA processing and modification	0	0	0
K	Transcription	160 [6.32]	174 [7.12]	196 [6.56]
L	Replication, recombination, and repair	126 [4.98]	125 [5.11]	128 [4.28]
B	Chromatin structure and dynamics	1 [0.04]	2 [0.08]	1 [0.03]
**Cellular processes and signalling**				
D	Cell cycle control, cell division, chromosome partitioning	21 [0.83]	23 [0.94]	25 [0.84]
Y	Nuclear structure	0	0	0
V	Defence mechanisms	15 [0.59]	31 [1.27]	27 [0.90]
T	Signal transduction mechanisms	109 [4.31]	71 [2.91]	128 [4.28]
M	Cell wall/membrane/envelope biogenesis	55 [2.17]	58 [2.37]	80 [2.68]
N	Cell motility	29 [1.15]	17 [0.70]	41 [1.37]
Z	Cytoskeleton	0	0	0
W	Extracellular structures	0	0	0
U	Intracellular trafficking, secretion, and vesicular transport	17 [0.67]	17 [0.70]	16 [0.54]
O	Posttranslational modification, protein turnover, chaperones	82 [3.24]	90 [3.68]	100 [3.35]
**Metabolism**				
C	Energy production and conversion	172 [6.80]	148 [6.06]	169 [5.65]
G	Carbohydrate transport and metabolism	83 [3.28]	64 [2.62]	74 [2.48]
E	Amino acid transport and metabolism	247 [9.76]	258 [10.56]	248 [8.30]
F	Nucleotide transport and metabolism	71 [2.81]	64 [2.62]	69 [2.31]
H	Coenzyme transport and metabolism	106 [4.19]	132 [5.40]	128 [4.28]
I	Lipid transport and metabolism	64 [2.53]	62 [2.54]	78 [2.61]
P	Inorganic ion transport and metabolism	133 [5.26]	133 [5.44]	138 [4.62]
Q	Secondary metabolites biosynthesis, transport, and catabolism	42 [1.66]	37 [1.51]	43 [1.44]
**Poorly characterized**				
R	General function prediction only	0	0	0
S	Function unknown	460 [18.18]	405 [16.57]	513 [17.16]
**All proteins**		2530	2444	2989
